# A rare case of splenic arteriovenous fistula causing portal hypertension, treated by embolisation

**DOI:** 10.4102/sajr.v30i1.3345

**Published:** 2026-02-12

**Authors:** Dane J. Rampini, John A. Cantrell

**Affiliations:** 1 Department of Interventional Radiology, Wits Donald Gordon Medical Centre, Johannesburg, South Africa; 2 Faculty of Health Sciences, University of the Witwatersrand, Johannesburg, South Africa

**Keywords:** splenic arteriovenous fistula, non-cirrhotic portal hypertension, vascular malformation, percutaneous embolisation, endovascular

## Abstract

This case report highlights the findings and treatment in a patient with a splenic arteriovenous fistula causing non-cirrhotic portal hypertension (NCPH). Splenic arteriovenous malformations (AVMs) are rare, and their management using percutaneous embolisation is an emerging alternative modality as opposed to open surgical intervention.

**Contribution:**

Similar cases have been published; however, few highlight the management role of interventional radiology.

## Introduction

Portal hypertension is most commonly associated with cirrhotic liver disease; however, non-cirrhotic portal hypertension (NCPH) represents an important differential diagnosis. Non-cirrhotic portal hypertension is caused by a heterogeneous group of disorders affecting the hepatic and portal venous circulation, with sites of resistance classified as pre-hepatic, intra-hepatic or post-hepatic.^1^ Clinical manifestations overlap significantly with cirrhotic portal hypertension, often leading to delayed or missed diagnosis. Splenic arteriovenous fistulas (SAVFs) are a rare pre-hepatic cause of NCPH in which high-flow shunting increases portal venous pressures. The condition is frequently under-recognised because of its rarity.^2^

Splenic arteriovenous fistulas may be congenital or acquired. Diagnosis requires a high index of suspicion, the use of multimodality imaging and confirmatory angiography.^1^ Management strategies include conservative, surgical or endovascular treatment. Historically, surgical resection with or without splenectomy was considered the mainstay of treatment; however, endovascular therapy using embolisation coils has become the preferred approach. Although endovascular techniques are associated with higher revascularisation rates, embolisation remains the preferred approach because of its minimally invasive nature and reduced procedure-related morbidity.^3^

This report describes a 59-year-old woman with SAVF and associated portal hypertension, successfully managed with endovascular embolisation. The case adds to the growing body of evidence supporting endovascular treatment while highlighting the importance of careful follow-up and consideration of adjunctive embolic agents to improve long-term outcomes.

## Patient presentation

A 59-year-old woman with a 3-week history of non-specific abdominal discomfort, abdominal distension, portal vein thrombosis seen on ultrasound and a normal liver biopsy was referred to the Wits Donald Gordon Medical Centre Radiology practice for further workup. The patient had no significant previous medical or surgical history. Laboratory investigations revealed a microcytic hypochromic anaemia with an Hb of 9.8 g/dl. Renal biochemistry, liver function tests and international normalised ratio (INR) were normal.

**FIGURE 1:**
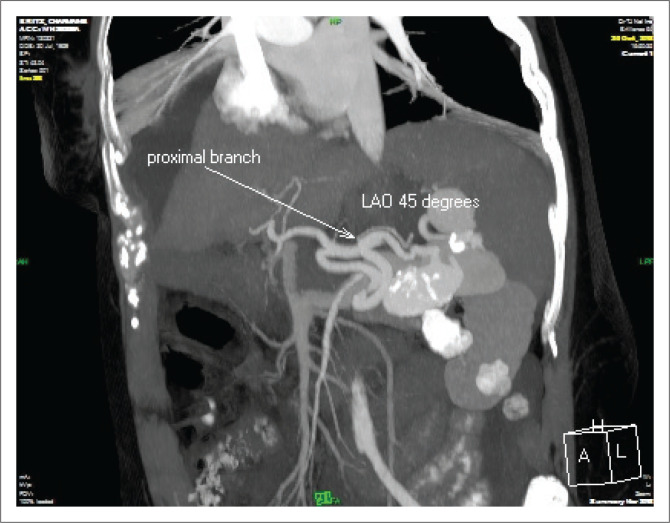
A triphasic liver CT scan displaying a large splenic artery to vein fistula.

An initial triphasic liver protocol CT scan revealed a large splenic artery to vein fistula (Figure 1), causing portal hypertension, ascites and dilation of both the splenic and portal veins.

The large distal splenic artery saccular aneurysm measured 48 mm × 29 mm in axial dimensions. The spleen was poorly vascularised and normal in size. Secondary to the fistula there was dilation of the splenic vein, measuring 13 mm in diameter. Extensive mural thrombus of the portal vein (narrowest residual lumen approximately 5 mm) extending into the intra hepatic portal veins and numerous small collaterals at the porta hepatis and gallbladder were also noted. No definite hepatic cirrhosis was evident.

**FIGURE 2:**
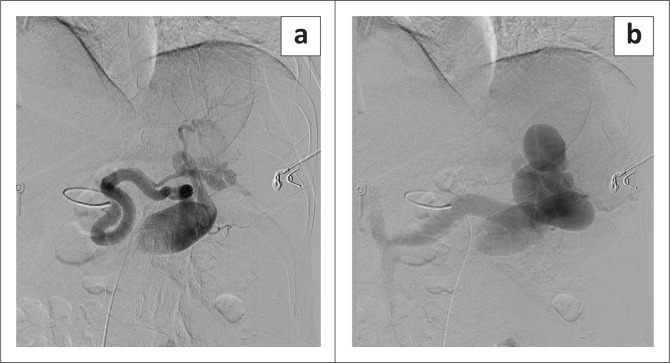
(a, b) Angiography demonstrating the large fusiform splenic artery aneurysm, and the aneurysmal dilation of the arterio-portal fistula at the splenic hilum, with relative devascularisation and early filling of the splenic and portal veins.

The patient was subsequently referred to interventional radiology. She was counselled and consented for splenic angiography and embolisation. General anaesthesia was administered. A five French sheath was inserted into the right common femoral artery under ultrasound guidance. A four French C2 catheter was inserted into the coeliac axis. Angiography demonstrated the large fusiform splenic artery aneurysm, and the aneurysmal dilation of the arterio-portal fistula at the splenic hilum, with relative devascularisation and early filling of the splenic and portal veins (Figure 2a and Figure 2b).

**FIGURE 3:**
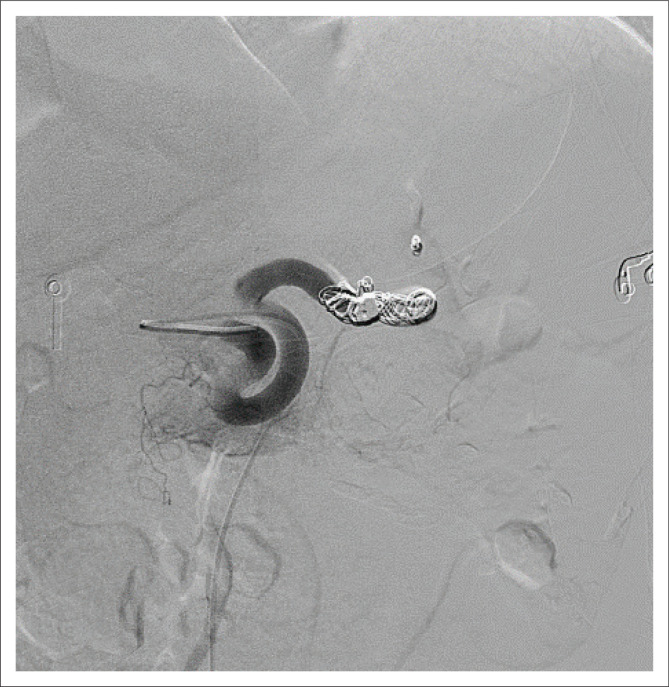
Angiography with complete occlusion of the splenic artery and no filling of the aneurysm or arterio-portal fistula.

A micro catheter was inserted into the splenic artery, and the tip positioned immediately proximal to the aneurysm. The splenic artery was embolised using six detachable haemostatic coils. Repeat angiography showed filling of the fistula via a small proximal splenic artery branch. This branch was catheterised with a micro catheter and embolised using three small haemostatic coils (2 mm – 3 mm diameter). Following a short interval of time for thrombosis, repeat angiography revealed a good technical result, with complete occlusion of the splenic artery and no filling of the aneurysm or arterio-portal fistula (Figure 3). The femoral puncture site was compressed for 10 min, and haemostasis was achieved.

A repeat triphasic liver CT on day 2 post embolisation demonstrated re-established flow within the splenic artery, past the haemostatic coils, filling the aneurysm and arterioportal fistula. The patient was counselled along with her referring physician, and the decision was made to repeat the angiogram with possible repeat embolisation.

**FIGURE 4:**
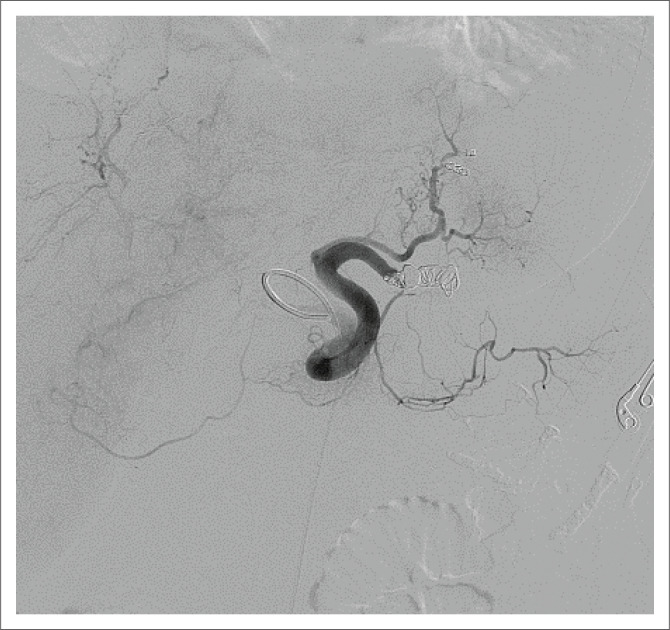
An indirect portal venogram demonstrating no flow in the splenic vein.

The patient returned to theatre on day 14 post initial embolisation. The splenic artery angiogram indicated occlusion of the distal splenic artery by the original haemostatic coils. Two small collateral arteries were visualised supplying the spleen but not feeding the splenic artery aneurysm or fistula. An indirect portal venogram was performed via the superior mesenteric artery, which demonstrated no flow in the splenic vein (Figure 4). Intra-procedural ultrasonography of the aneurysm and dilated fistula showed thrombosis of both structures, as well as thrombosis of the lateral aspect of the splenic vein. It was concluded that the flow dynamics were ideal, and no further embolisation was performed.

## Discussion

Non-cirrhotic portal hypertension, also known as idiopathic non-cirrhotic portal hypertension (INCPH), is characterised by increased portal venous pressures without the presence of liver disease or portal vein thrombosis.^2^ Non-cirrhotic portal hypertension can be classified according to anatomical sites of resistance to blood flow as pre-hepatic, intra-hepatic and post-hepatic. Hepatic classification can be further divided into pre-sinusoidal, sinusoidal and post-sinusoidal.^1^ Presenting patients commonly show typical signs of portal hypertension, such as variceal bleeding, splenomegaly, caput medusae and hepatic encephalopathy. Diagnosis of NCPH is complex as there is no single diagnostic investigation. Diagnosis is thus based on a combination of clinical evaluation, radiological imaging and the exclusion of classical cirrhotic liver disease through biopsy.^4^ It is theorised that up to 10% of cases with portal hypertension are related to NCPH.^5^

Splenic arteriovenous fistulas are a rare cause of pre-hepatic NCPH. These fistulas have a double mechanism of action in causing portal hypertension by increasing both the volume and pressure in the hepatic portal vein. This further results in secondary fibrosis of small hepatic vein branches and loss of sinusoidal basement membrane fenestrations (capillarisation), adding an intra-hepatic element.^6^ A report by Lomeli and Gorospe in 2013, documented less than 50 cases of NCPH as a result of SAVF.^7^

Causes of SAVF are either congenital or acquired, post-traumatic, pre-existing splenic artery aneurysm rupture, post infectious causes or as complications of splenectomy.^7^ Diagnosis is commonly missed unless there is a high index of suspicion. Doppler ultrasound, contrasted CT and MRI with multiple phases are quick and non-invasive methods for detecting vascular abnormalities; however, direct angiography of the coeliac axis or mesenteric artery is often required for a definitive diagnosis.^8,9^

Three major treatment options may be considered: conservative management, open repair and endovascular repair. Open surgical repair consists of resection of the vascular anomaly, with or without splenectomy, aimed at reducing the risk of morbidity and mortality because of malformation rupture rather than managing the portal hypertension.^10^ Endovascular management consists mainly of the use of endovascular coils and has become increasingly popular and accepted in recent years.

A large systematic review published in 2014 compared the three treatment options and concluded that endovascular repair had better short-term outcomes compared to open surgery but was also associated with an increased frequency of late complications and greater rate of re-interventions. Conservative treatment was associated with higher rates of mortality.^3^

Endovascular repair with coils remains the mainstay of percutaneous management; however, evidence suggests the use of adjuncts may be beneficial. Alexander and Santos highlighted the use of coils and n-butyl cyanoacrylate glue, suggesting that coils act as a scaffold to slow arterial flow and for the n-butyl cyanoacrylate glue to attach, causing near-instant vascular occlusion. The theory is that this dual treatment would create a more durable occlusion with faster results and a decreased need for re-intervention.^11^

In the presented case, initial treatment consisted of endovascular coils; however, follow-up CT investigations revealed re-established flow within the splenic artery, beyond the haemostatic coils, filling the aneurysm and arterioportal fistula. Augmentation of the embolisation with a glue medium may have accelerated the time for full fistula embolisation to be established and negated the need for repeat direct angiography.

## Conclusion

Splenic arteriovenous fistulas are an uncommon, but clinically significant cause of NCPH. Their rarity and non-specific presentation often result in delayed diagnosis.

Endovascular embolisation is now considered the mainstay of therapy as a result of its safety profile and minimally invasive approach. However, as demonstrated in this case, coil embolisation alone may not always achieve immediate occlusion, particularly in large or complex fistulas associated with aneurysmal change. Adjunctive techniques may improve long-term success and reduce the need for re-intervention.

This case highlights the value of multidisciplinary management and strict follow-up in ensuring optimal outcomes.

By sharing this case, we aim to increase awareness of SAVFs as a rare but treatable cause of portal hypertension and contribute to the evolving discussion on the best therapeutic strategies for these challenging vascular lesions.

## Learning points and highlights

- Splenic arteriovenous malformations (AVMs) are a rare but treatable cause of portal hypertension.
- Non-cirrhotic portal hypertension should prompt a thorough vascular evaluation.
- Endovascular embolisation or splenectomy can be curative.
- Misdiagnosis can lead to unnecessary treatments for cirrhosis.
